# Supplementary material for: Extended X-ray absorption fine structure of dynamically-compressed copper up to 1 terapascal
Source: Nat Commun. 2023 Nov 10;14:7046. doi: 10.1038/s41467-023-42684-7 (PMC10638371; doi:10.1038/s41467-023-42684-7)
Supplement: Supplementary file 1 — Supplementary Information [file 41467_2023_42684_MOESM1_ESM.pdf]

Supplementary Information

Extended X-ray absorption fine structure of dynamically-compressed copper up to 1 terapascal

SUPPLEMENTARY NOTE 1

The experimentally-determined pressure, density, temperature, and phase of all experiments discussed in the manuscript is summarized here. Pressure is determined from characteristic analysis of velocimetry measurements. Density, temperature, and phase are determined from EXAFS measurements. Analysis details are presented in subsequent sections.

Supplementary Table 1: Cu EXAFS experiment summary. Experimentally-determined pressure, density, and temperature near 400 GPa and near 1 TPa.

Shot	Adjacent layers	Cu phase	Pressure GPa	Density g cm^{-3}	Temperature K
N200520-001	Be/**Cu**/diamond	fcc	414^{+52}_{-15}	16 ± 0.4	3900 ± 400
N200917-001	Al/**Cu**/LiF	fcc	390 ± 20	15.8 ± 0.4	3200 ± 400
N201222-001	Al/**Cu**/LiF	fcc	453 ± 20	16.4 ± 0.3	2300 ± 300
N210719-002	diamond/**Cu**/diamond	fcc	430^{+52}_{-15}	15.5 ± 0.4	5600 ± 500
N211127-001	diamond/**Cu**/diamond	indeterminate	1053^{+55}_{-20}	20.2 ± 1.5	8500 ± 1500

SUPPLEMENTARY NOTE 2

Simultaneous velocimetry measurements of the Cu/window interface and/or the window free surface are made alongside the EXAFS measurements using a VISAR (Velocity Interferometer System for Any Reflector)[1] system. The NIF dual-channel, line-imaging VISAR system detects Doppler shifts of a 660-nm optical probe reflecting off a moving surface (in these experiments, either the Cu-window interface or the window free surface). The reflected signal is transmitted through a Mach-Zehnder interferometer, and the Doppler shift is encoded in the phase of the interference fringes. The first leg of the VISAR used an etalon thickness of 19.98 mm, corresponding to a vacuum velocity per fringe of 3.125 km s^{-1}. The second leg of the VISAR used an etalon thickness of 49.99 mm, with a vacuum velocity per

fringe of 1.249 km s^{-1}. An angled mirror (25-μm thick and made from black nano-diamond polished to optical quality) is positioned behind the target to reflect optical light from the back of the target to the VISAR. This thin diamond window does not interfere with the EXAFS measurement as its X-ray transmission is smooth in the EXAFS region.

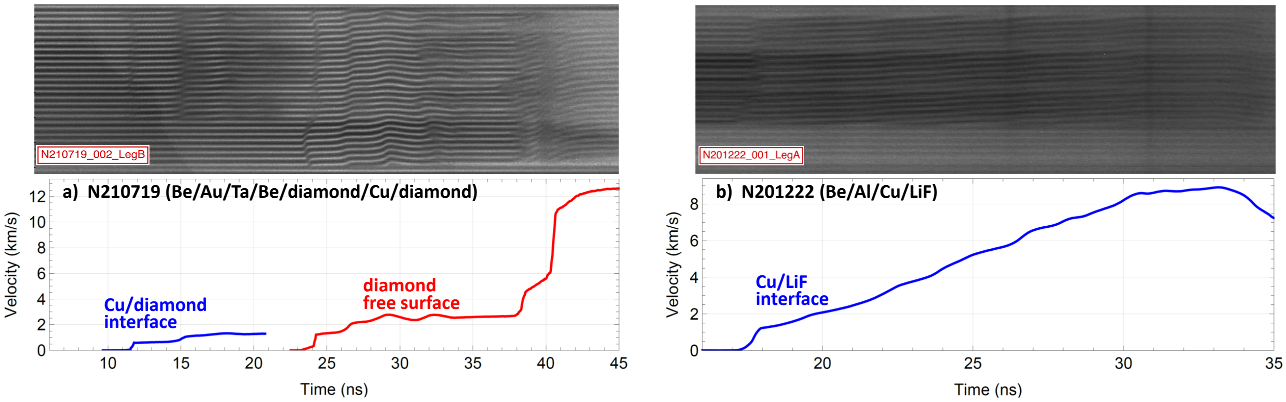

Supplementary Figure 1: VISAR data examples. **a** VISAR data and velocities (at the Cu/diamond interface, blue, and at the diamond free surface, red) for N210719. **b** VISAR data and velocity (at the Cu/LiF interface, blue) for N201222. Velocity at the Cu/diamond interface and at the Cu/LiF interface has been corrected[2, 3]. Source data are provided as a Source Data file.

Representative VISAR data and velocities at the Cu/window interface and/or window free surface are shown in Supplementary Fig. 1. In targets with a diamond window (Supplementary Fig. 1, left), Cu/diamond interface velocity at early times and diamond free surface velocity are measured (diamond becomes opaque when compressed above the Hugoniot elastic limit at ∼80 GPa[4]). In target with a LiF window (Supplementary Fig. 1, right), the apparent velocity at the Cu/LiF interface is measured, from which the particle velocity of the interface is obtained using the density-dependence of the index of refraction of LiF under ramp-compression[2]. The apparent velocity at the Cu/diamond interface is also corrected[3]. The diamond and LiF windows have a Ti flash coating covering 1/3 of their rear surface (corresponding to the bottom third of the images in Supplementary Fig. 1). Therefore the observed fringe motion in that region corresponds to compression waves breaking out of the rear surface.

The Cu pressure at the time of EXAFS measurement is determined through the measured velocities at the Cu/window interface and/or window free surface using a backward hydrodynamic characteristic analysis[5, 6]. For example, for targets with a diamond win-

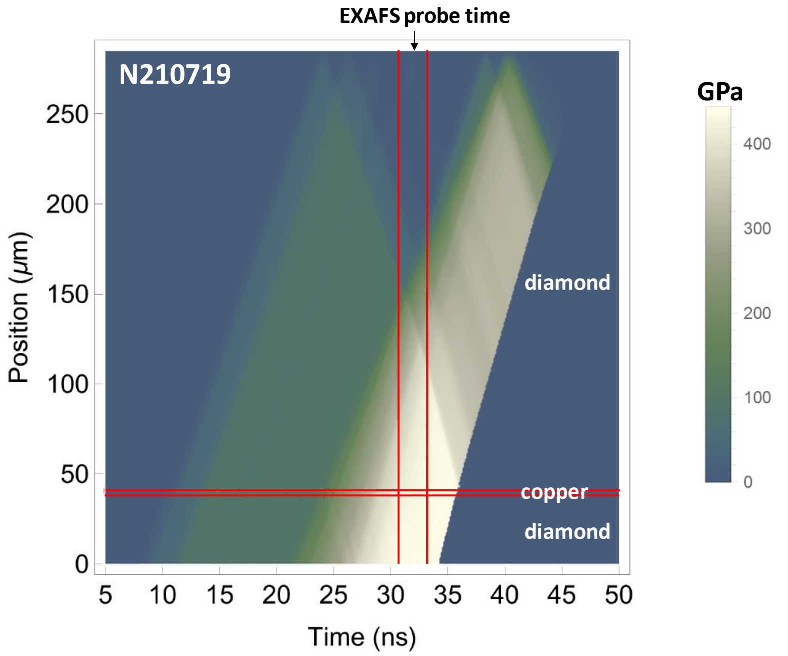

Supplementary Figure 2: Characteristic Analysis example. Pressure profile in the diamond-Cu-diamond layers in the target for N210719 determined from characteristic analysis. The horizontal red lines denote the boundaries of the Cu layer, and the vertical red lines denote the EXAFS probe duration.

dow, the diamond free surface velocity serves as a boundary condition where material flow is interpreted as a series of positive and negative characteristics. Pressure waves are back-propagated through the window from the free surface to the Cu layer using the window's equation of state. It is important to note that an accurate knowledge of the Cu equation of state is not essential for determining the Cu sample pressure because the diamond behaviors under compression (using the experimentally measured values in Bradley *et al.*[7]) dominates the response of the target, especially if the Cu layer is thin, as the Cu pressure will equilibrate with adjacent layers. Supplementary Figure 2 shows an example of characteristic analysis pressure profile in the diamond-Cu-diamond layers in the target for N210719. The final uncertainty in the Cu pressure is a combination of the velocity uncertainty (∼2%-3%) and spatial/temporal gradients in the Cu sample during EXAFS probe. One additional source of uncertainty in the characteristic analysis framework related to the diamond window is the assumption that diamond follows a reversible isentropic path upon compression and release. This leads to a possible 50-GPa stress underestimate[8] represented by asymmetric uncertainties.

SUPPLEMENTARY NOTE 3

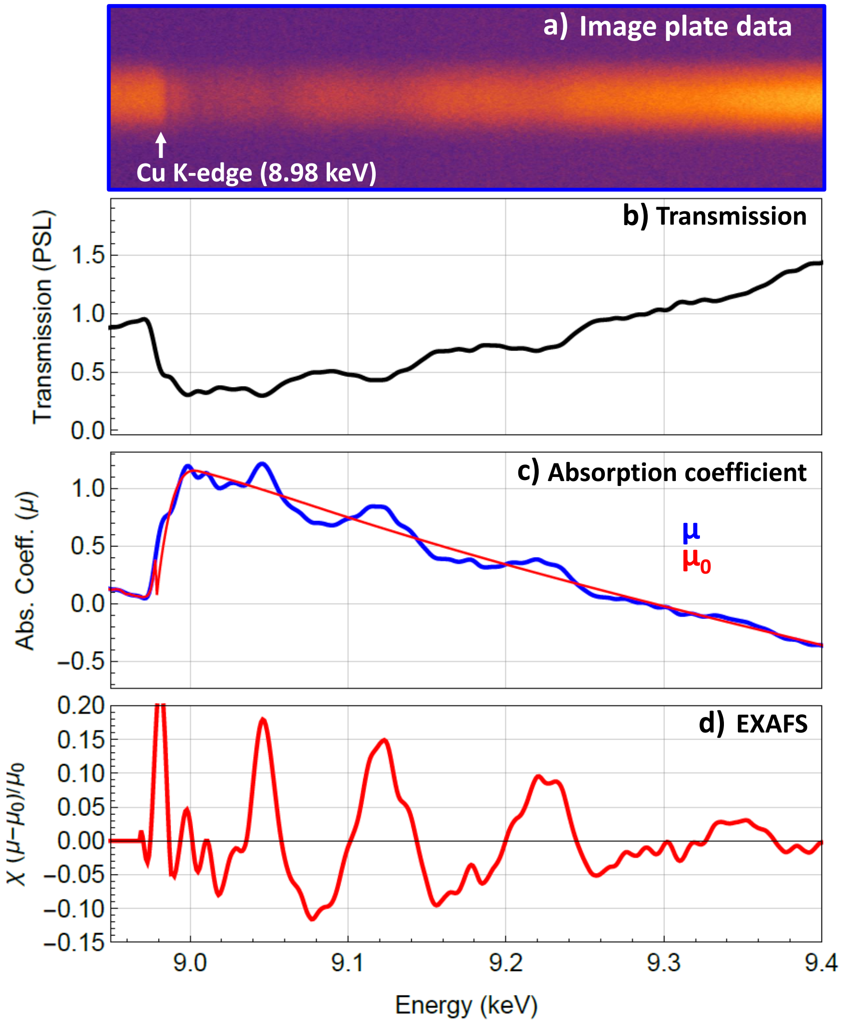

Supplementary Figure 3: EXAFS data processing. Data processing flow of EXAFS data from **(a)** image plate data, to **(b)** transmission (black), to **(c)** absorption coefficient (blue) with background (red) subtraction, and finally to **(d)** EXAFS oscillations (red). Source data are provided as a Source Data file.

This section provides an overview of EXAFS data processing and sources of uncertainty. X-ray transmission data from the HiRAXS[9] spectrometer is recorded on a Fuji SR image plate[10], and a representative image plate data is shown in Supplementary Fig. 3a. The vertical extent of the EXAFS signal is $\sim$ 80-100 50-μm pixel. The energy dispersion of the HiRAXS is $\sim$ 1 eV per 50-μm pixel. The resolution of the HiRAXS spectrometer in this configuration is typically $\sim$ 3-4 eV. SR image plate sensitivity to 9-keV photons is 2.6 mPSL per photon[10]. This translates to a photon statistics of $\sim$ 240 PSL per resolution element,

or 9×10^4 X-ray photons (N) per resolution element, corresponding to a photon statistics $\sqrt{N}/N \sim 0.3\%$. This estimate is in good agreement with the observed noise level in the X-ray transmission. The EXAFS signal region (center) is background subtracted using the regions immediately above and below. The magnitude of the Cu edge jump in PSL unit is typically ~0.5-1.0 PSL per 50-μm pixel. A Savitzky-Golay filter is used to smooth the data over one resolution element before proceeding to the next step.

The energy axis of the X-ray transmission is established using the two visible K-edges on the image plate (Cu at 8.979 keV and Zn at 9.659 keV) and the calculated energy dispersion of the HiRAXS spectrometer. The measured X-ray transmission is converted to absorption coefficient μ and then EXAFS signal χ using the software package ATHENA[11]. Once the X-ray transmission has been converted to X-ray absorption coefficient μ, the regions before and after the K-edge are fitted and extrapolated to the K-edge. Then, the signal is normalized such that the edge jump is unity. A spline is fitted to the absorption coefficient to represent background non-oscillatory portion of μ (μ_0 in Supplementary Fig. 3c). At this point, the EXAFS oscillations is extracted as $\chi = (\mu - \mu_0)/\mu_0$.

The final uncertainty in the EXAFS peak amplitudes is a combination of the noise in the X-ray transmission measurement ($\sim 0.3\%$) and variance in the background subtraction process. We have found that our measured Cu K-edge EXAFS data is quite insensitive to analysis choices made during normalization and spline-fitting, and estimate the uncertainty in the EXAFS peak amplitude to be $\sim 0.5\% - 1.0\%$.

SUPPLEMENTARY NOTE 4

The commissioning of the NIF EXAFS spectrometer (HiRAXS[9]) began with Cu EXAFS measurement of a Cu foil at the entrance of the HiRAXS spectrometer 252 mm away from the Ti X-ray source. At this distance, the Cu foil is expected to remain in ambient conditions.

Before proceeding with driven EXAFS experiment, it is important to quantify the heating introduced by the Ti X-ray source in the Cu sample. In the second experiment, the Cu sample is placed in the target package much closer to the Ti X-ray source (25 mm). Both the target package and Ti X-ray source are in the same configuration as used in a driven EXAFS experiment.

We compare the EXAFS peak amplitudes between ambient Cu EXAFS (Supplementary

Fig. 4, blue) and undriven Cu EXAFS (Supplementary Fig. 4, red) using the the ratio method[12], and constrain the Ti X-ray source heating of the Cu sample to less than 200 K. This estimate is independently confirmed by calculating the X-ray source spectrum transmission through different layers in the target package and the X-ray absorption in the Cu sample, as well as FEFF calculations using a Debye model.

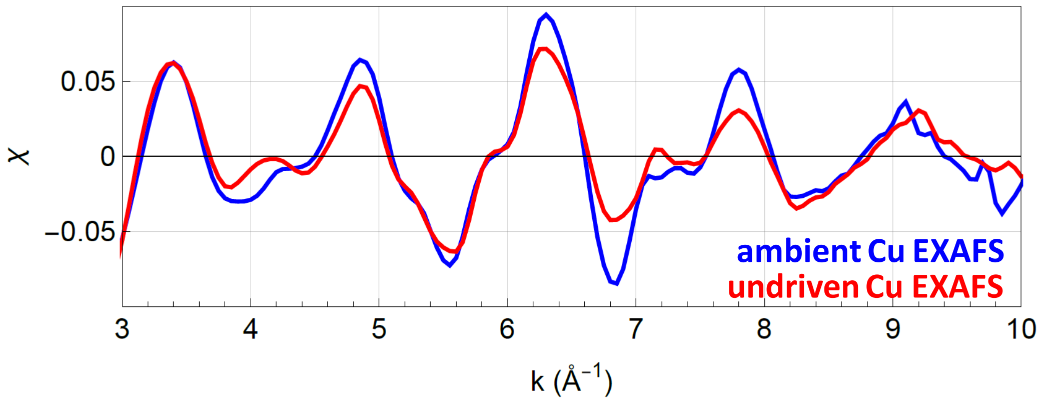

Supplementary Figure 4: Ambient and undriven EXAFS. Cu EXAFS measured in ambient conditions (N200111-001, blue) and Cu EXAFS from a Cu sample in an undriven target (N200519-001, red). Source data are provided as a Source Data file.

SUPPLEMENTARY NOTE 5

For the classical Molecular Dynamic (MD) simulations, synthetic EXAFS are calculated from snapshots of the atomic structure from equilibrium MD simulations of a perfect fcc (or bcc) copper lattice at the specific density and temperature using the MD code Large-scale Atomic/Molecular Massively Parallel Simulator (LAMMPS)[13] with the Mishin potential[14]. A large cluster ($\sim$ 300,000 atoms) is used.

To validate the accuracy of the classical MD simulations, we carried out finite-temperature density-functional-theory Molecular Dynamics (DFT-MD) simulations. They were performed using the Vienna *ab initio* simulation package (VASP)[15–17] within the PBE-GGA exchange-correlation functional[18] (and tests within PBEsol[19]), using 256 (for fcc and liquid) and 250 (for bcc) atom supercells, a k-point at (1/4,1/4,1/4) , an 11- and a 19-electron PAW pseudopotentials near 400 GPa and 1 TPa, respectively, a 500 eV plane wave cut off, a Nose-Hoover thermostat, and a 2.0 fs ionic time step.

For MD simulation output (one time snapshot of a large cluster of $\sim$ 300,000 atoms), a

randomly selected atom cluster with a radius of 8 $\mathring{A}$ is used as input configuration for one FEFF calculation. This process is repeated 500 times and the FEFF outputs are averaged. For DFT-MD simulations (many time snapshots of 256 atoms), the same process is used, except each FEFF calculation instead corresponds to a different time snapshot. We check convergence by confirming that the result does not change when adding more configurations. We used a path length parameter of 6 $\mathring{A}$ and included paths up to 4 scattering legs, and have tested sensitivity on the EXAFS outputs for different path lengths and scattering legs. These calculations used a curved-wave and plane-wave cutoffs of 5.0% and 3.5%, respectively.

A comparison of synthetic EXAFS generated from MD and DFT-MD simulations near 400 GPa is shown in Supplementary Fig. 5a for two different temperatures. Supplementary Fig. 5b shows how the first shell second cumulant (σ^2) changes as a function of temperature, and there is very good agreement between MD and DFT-MD simulations. The overall good agreement between MD and DFT-MD simulations is important, as there is no reliable measurement of Debye temperature in the pressure range of interest, and the correlated Debye model is expected to be increasingly inaccurate for $T \gg T_{Debye}$[20].

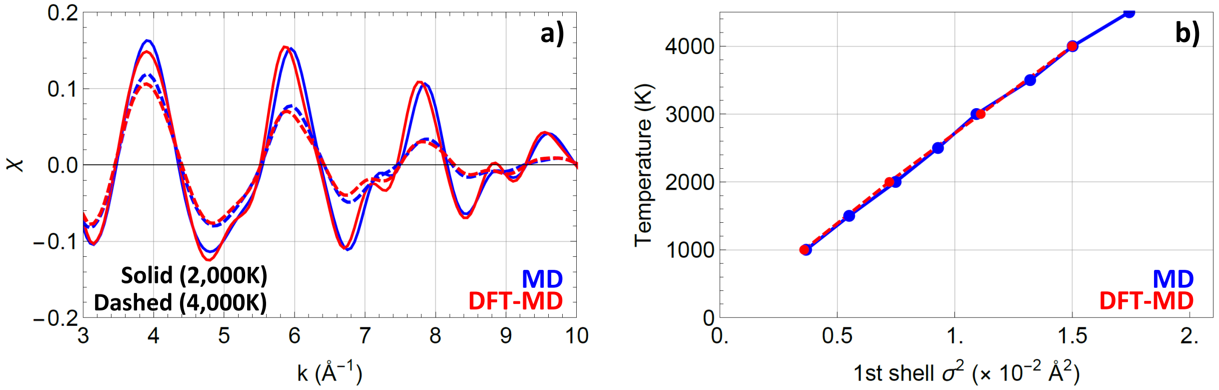

Supplementary Figure 5: MD and DFT-MD simulations. **a** synthetic MD (blue) and DFT-MD (red) EXAFS at 2,000 K (dashed) and 4,000 K (solid) for an fcc Cu lattice with atomic volume of 6.4 $\mathring{A}^3$ (near 400 GPa). **b** calculated second cumulant (1st shell) from atomic positions.

Supplementary Fig. 6 shows the density and temperature dependence of Cu EXAFS near 16.5 g cm^{-3} and 2000 K using synthetic EXAFS calculated from MD simulations. To begin the discussion on Cu EXAFS sensitivity to density and temperature, we note that density affects both EXAFS peak amplitudes and positions, whereas temperature primarily only affects peak amplitudes. Supplementary Figure 6a shows that a density change of 0.5

g cm^{-1} gives a noticeable shift in peak positions, and in practice, we found the density uncertainty is $\sim$ 0.3-0.4 g cm^{-3}. In mapping measured EXAFS data to a density and temperature using MD simulations, density and temperature are varied until a best match to the measured EXAFS data is found by minimizing the difference between measured and synthetic EXAFS.

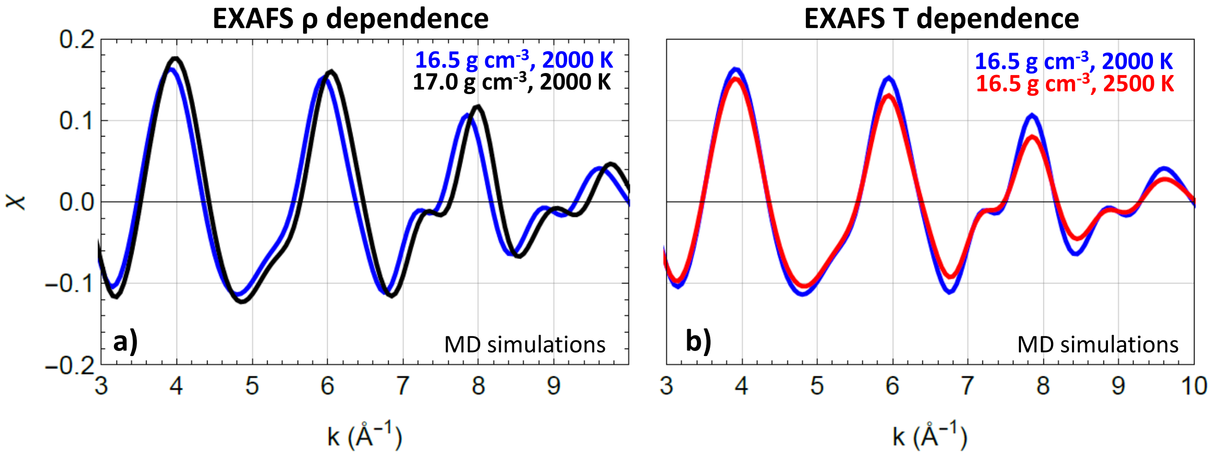

Supplementary Figure 6: EXAFS density and temperature dependence. Synthetic EXAFS calculated from MD simulations at **(a)** different densities (blue, black), and **(b)** different temperatures (blue, red).

As an example, Supplementary Fig. 7a shows the measured EXAFS on N201222 as described in the manuscript, and the shaded area indicates the spread in synthetic EXAFS from MD simulations given a temperature spread of $\pm$ 300 K. Supplementary Figure 7b shows how the total fit residual squared varies as a function of MD density and temperature. The total fit residual squared is defined as the difference between measured EXAFS and synthetic EXAFS (at a specific MD density and temperature), squared, and summed. The final EXAFS temperature uncertainty is a combination of peak amplitude uncertainty and EXAFS temperature sensitivity (less sensitive at higher temperature). This approach naturally captures and includes anharmonicity in the atomic distribution.

We have also independently investigated the effect of anharmonicity by instead fitting the measured EXAFS signals to multiple scattering paths assuming a fcc lattice (the same approach as used in Ref. [20]). This enables explicitly including or excluding the third cumulant (skewness) of the first shell as a fitting parameter and noting its effects on the first cumulant (relating to density) and second cumulant (relating to temperature). Overall, we find that explicitly including or excluding an anharmonic fitting parameter (skewness) in

a fit to multiple scattering paths did not lead to noticeably different results outside fitting uncertainty (near 400 GPa, up to 6,000 K). There is a more noticeable effect near 1 TPa ($\sim$ 9,000 K). However, parameter uncertainties are also larger for the 1-TPa experiment, so the difference with and without anharmonicity is still within uncertainty.

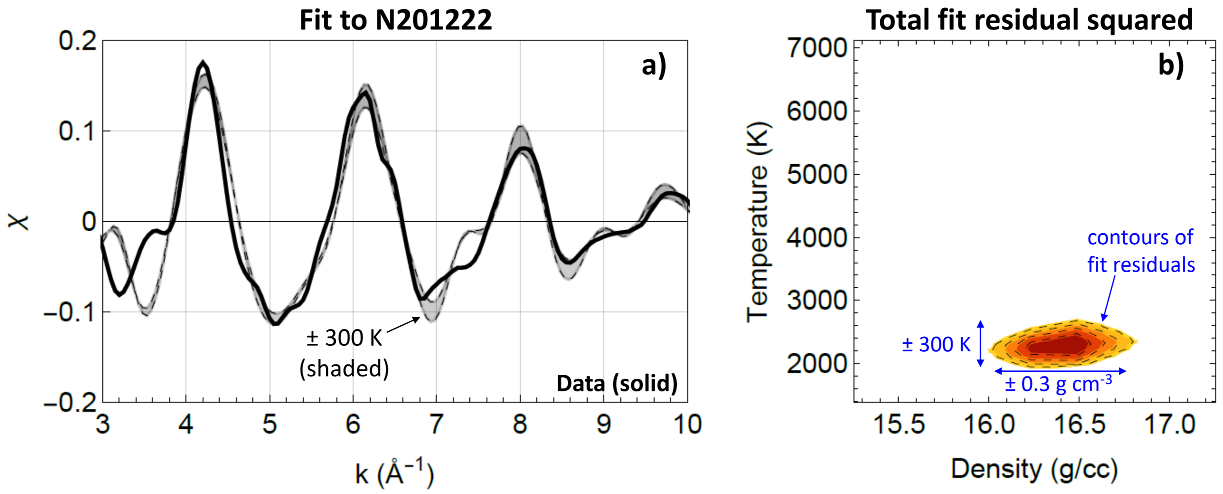

Supplementary Figure 7: EXAFS fitting. **a** MD fit to N201222. Data is represented by the black solid line, and the black shaded area indicates the spread in synthetic EXAFS from MD simulations given a temperature spread of $\pm$ 300 K. **b** Contour plot of total residual squared between measured data and MD simulations at different densities and temperatures, with the shading indicating regions of different total fit residual squared.

SUPPLEMENTARY NOTE 6

We first clarify the impact of measurement resolution on the interpretation of near-edge data measured on NIF. Supplementary Figure 8 shows the Cu K-edge data (near-edge) in ambient conditions taken at NIF (this work) and at a synchrotron facility with $<$ 1 eV resolution for comparison. To determine the spectral resolution, the synchrotron XANES spectrum is blurred by a Gaussian function with various Full-Width-Half-Maximum (FWHM) to compare with our undriven data. We found that a FWHM = 4 eV matches the undriven data best (Supplementary Fig. 8). While the spectrometer can achieve a resolution of 3 eV[9], we used a configuration with a larger crystal area to increase signal to noise, which slightly degraded resolution.

As we see in Supplementary Fig. 9a, this measurement resolution has a small but noticeable effect on the near-edge structure, and may blur out small differences in the near-edge

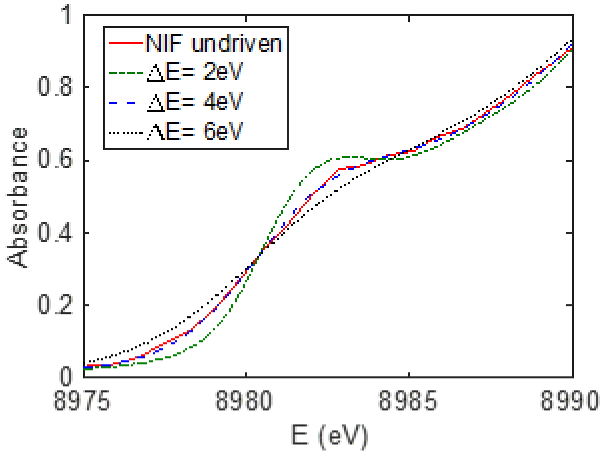

Supplementary Figure 8: Measurement resolution. Cu K-edge data (near-edge) in undriven conditions taken at the NIF (this work, red) and at a synchrotron facility blurred with a Gaussian function with different FWHM (2 eV, green, 4 eV, blue, and 6 eV, black).

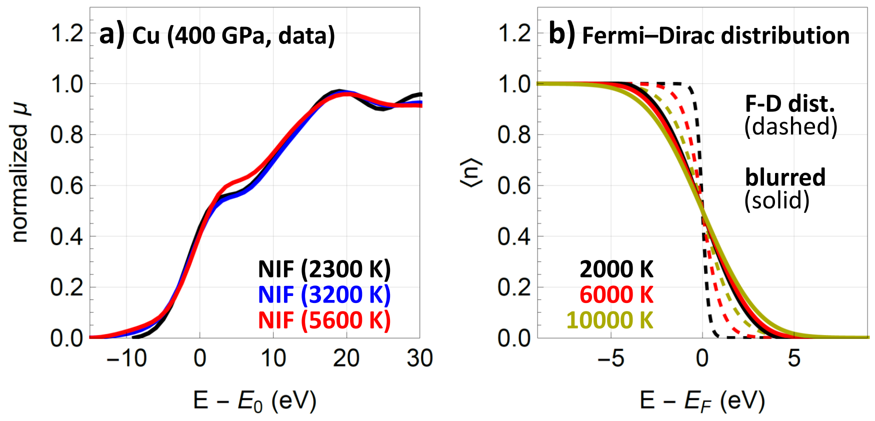

Supplementary Figure 9: Cu K-edge structure. **a** Cu K-edge data (near-edge) of Cu samples compressed to 400 GPa at 2,300 K (black), 3,200 K (blue), and 5,600 K (red). **b** the Fermi-Dirac distribution at different temperatures (dashed), and when blurred over the measurement resolution (solid). The Fermi-Dirac distribution at different temperatures of 2,000 K, 6,000 K, and 10,000 K are represented by black, red, and yellow, respectively. Here, E_0 is the Cu K-edge, and E_f is the Fermi energy. Source data are provided as a Source Data file.

from temperature effect. This measurement resolution is already taken into account in analysis of all EXAFS data.

As the 400 GPa NIF data discussed in this work covers a relatively small temperature range (from 2,300 K to 5,600 K), we examine the expected broadening of the Fermi-Dirac distribution with increasing temperature. Supplementary Fig. 9b plots the Fermi-Dirac distributions at 2,000 K, 6,000 K, and 10,000 K (all dashed), and also when the distributions

are blurred by the measurement resolution (solid). We observe that the difference in the Fermi-Dirac distributions of different temperatures is difficult to distinguish with the NIF measurement resolution (4 eV) included. In experiments[21–23] at higher temperature (up to $\sim$ 20,000K) and higher measurement resolution ($\sim$ 1-2 eV), temperature sensitivity in the near-edge structure is more readily observed.

SUPPLEMENTARY REFERENCES

[1] P. M. Celliers and M. Millot, Imaging velocity interferometer system for any reflector (VISAR) diagnostics for high energy density sciences, Review of Scientific Instruments **94**, 011101 (2023).

[2] L. E. Kirsch, S. J. Ali, D. E. Fratanduono, R. G. Kraus, D. G. Braun, A. Fernandez-Pañella, R. F. Smith, J. M. McNaney, and J. H. Eggert, Refractive index of lithium fluoride to 900 gigapascal and implications for dynamic equation of state measurements, Journal of Applied Physics **125**, 175901 (2019).

[3] M. Knudson, J. Asay, S. Jones, and Y. Gupta, Shock response of diamond crystals, Sandia Report SAND2001-3838 (2001).

[4] R. S. McWilliams, J. H. Eggert, D. G. Hicks, D. K. Bradley, P. M. Celliers, D. K. Spaulding, T. R. Boehly, G. W. Collins, and R. Jeanloz, Strength effects in diamond under shock compression from 0.1 to 1 TPa, Phys. Rev. B **81**, 014111 (2010).

[5] J. R. Maw, A characteristics code for analysis of isentropic compression experiments, AIP Conference Proceedings **706**, 1217 (2004).

[6] S. D. Rothman, J.-P. Davis, J. Maw, C. M. Robinson, K. Parker, and J. Palmer, Measurement of the principal isentropes of lead and lead–antimony alloy to ~400 kbar by quasi-isentropic compression, Journal of Physics D: Applied Physics **38**, 733 (2005).

[7] D. K. Bradley, J. H. Eggert, R. F. Smith, S. T. Prisbrey, D. G. Hicks, D. G. Braun, J. Biener, A. V. Hamza, R. E. Rudd, and G. W. Collins, Diamond at 800 GPa, Phys. Rev. Lett. **102**, 075503 (2009).

[8] J. K. Wicks, R. F. Smith, D. E. Fratanduono, F. Coppari, R. G. Kraus, M. G. New-

man, J. R. Rygg, J. H. Eggert, and T. S. Duffy, Crystal structure and equation of state of Fe-Si alloys at super-Earth core conditions, Science Advances **4**, eaao5864 (2018), https://www.science.org/doi/pdf/10.1126/sciadv.aao5864.

[9] S. Stoupin, D. B. Thorn, N. Ose, L. Gao, K. W. Hill, Y. Ping, F. Coppari, B. Kozioziemski, A. Krygier, H. Sio, J. Ayers, M. Bitter, B. Kraus, P. C. Efthimion, and M. B. Schneider, The multi-optics high-resolution absorption x-ray spectrometer (HiRAXS) for studies of materials under extreme conditions, Review of Scientific Instruments **92**, 053102 (2021).

[10] M. J. Rosenberg, D. B. Thorn, N. Izumi, D. Williams, M. Rowland, G. Torres, M. Haugh, P. Hillyard, N. Adelman, T. Schuler, M. A. Barrios, J. P. Holder, M. B. Schneider, K. B. Fournier, D. K. Bradley, and S. P. Regan, Image-plate sensitivity to x rays at 2 to 60 keV, Review of Scientific Instruments **90**, 013506 (2019).

[11] B. Ravel and M. Newville, *ATHENA*, *artemis*, *hephaestus*: data analysis for x-ray absorption spectroscopy using *ifeffit*, Journal of Synchrotron Radiation **12**, 537 (2005).

[12] P. Fornasini, S. a Beccara, G. Dalba, R. Grisenti, A. Sanson, M. Vaccari, and F. Rocca, Extended x-ray-absorption fine-structure measurements of copper: Local dynamics, anharmonicity, and thermal expansion, Phys. Rev. B **70**, 174301 (2004).

[13] S. Plimpton, Fast parallel algorithms for short-range molecular dynamics, Journal of Computational Physics **117**, 1 (1995).

[14] Y. Mishin, M. J. Mehl, D. A. Papaconstantopoulos, A. F. Voter, and J. D. Kress, Structural stability and lattice defects in copper: Ab initio, tight-binding, and embedded-atom calculations, Phys. Rev. B **63**, 224106 (2001).

[15] G. Kresse and J. Hafner, Ab initio molecular dynamics for liquid metals, Phys. Rev. B **47**, 558 (1993).

[16] G. Kresse and J. Furthmüller, Software VASP, vienna (1999), Phys. Rev. B **54**, 169 (1996).

[17] G. Kresse and D. Joubert, Efficient iterative schemes for ab initio total-energy calculations using a plane-wave basis set. From ultrasoft pseudopotentials to the projector augmented-wave method, Physical Review B **59**, 1758 (1999).

[18] J. P. Perdew, K. Burke, and M. Ernzerhof, Generalized gradient approximation made simple (vol 77, pg 3865, 1996) (1997).

[19] J. P. Perdew, A. Ruzsinszky, G. I. Csonka, O. A. Vydrov, G. E. Scuseria, L. A. Constantin, X. Zhou, and K. Burke, Restoring the density-gradient expansion for exchange in solids and

surfaces, Phys. Rev. Lett. **100**, 136406 (2008).

[20] Y. Ping, F. Coppari, D. G. Hicks, B. Yaakobi, D. E. Fratanduono, S. Hamel, J. H. Eggert, J. R. Rygg, R. F. Smith, D. C. Swift, D. G. Braun, T. R. Boehly, and G. W. Collins, Solid iron compressed up to 560 GPa, Phys. Rev. Lett. **111**, 065501 (2013).

[21] N. Jourdain, L. Lecherbourg, V. Recoules, P. Renaudin, and F. Dorchies, Electron-ion thermal equilibration dynamics in femtosecond heated warm dense copper, Phys. Rev. B **97**, 075148 (2018).

[22] B. I. Cho, K. Engelhorn, A. A. Correa, T. Ogitsu, C. P. Weber, H. J. Lee, J. Feng, P. A. Ni, Y. Ping, A. J. Nelson, D. Prendergast, R. W. Lee, R. W. Falcone, and P. A. Heimann, Electronic structure of warm dense copper studied by ultrafast x-ray absorption spectroscopy, Phys. Rev. Lett. **106**, 167601 (2011).

[23] A. Mančić, A. Lévy, M. Harmand, M. Nakatsutsumi, P. Antici, P. Audebert, P. Combis, S. Fourmaux, S. Mazevet, O. Peyrusse, V. Recoules, P. Renaudin, J. Robiche, F. Dorchies, and J. Fuchs, Picosecond short-range disordering in isochorically heated aluminum at solid density, Phys. Rev. Lett. **104**, 035002 (2010).
